# First person – Tanner Robertson

**DOI:** 10.1242/dmm.052926

**Published:** 2026-04-02

## Abstract

First Person is a series of interviews with the first authors of a selection of papers published in Disease Models & Mechanisms, helping researchers promote themselves alongside their papers. Tanner Robertson is first author on ‘ Evidence that xylazine disrupts skin homeostasis by acting on epithelial cells through the kappa opioid receptor’, published in DMM. Tanner is a postdoctoral fellow in the lab of Anna Huttenlocher at University of Wisconsin School of Medicine and Public Health, Madison, WI, USA, investigating adaptive immunity and T-cell migration using zebrafish models.

**Figure:**
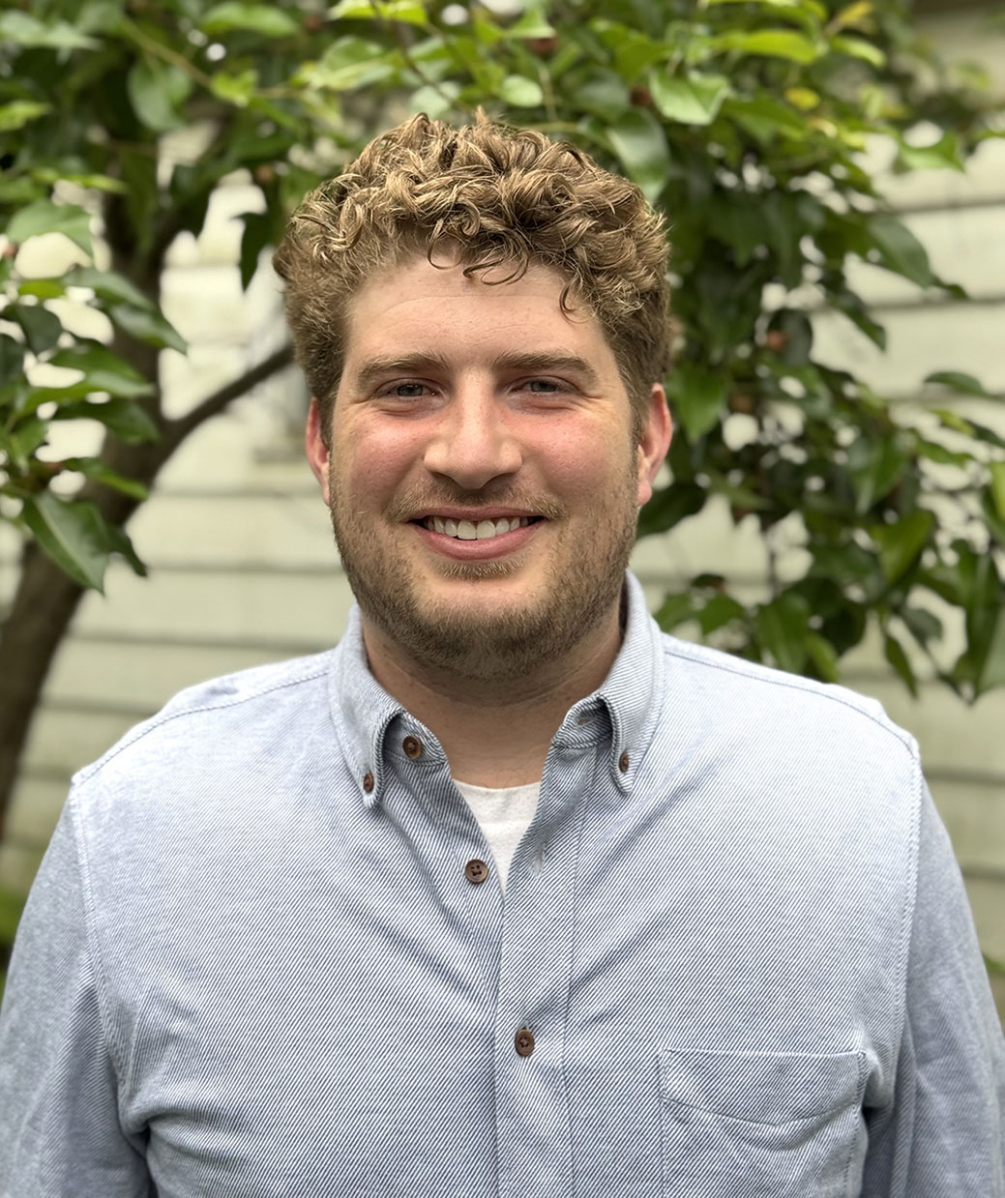
Tanner Robertson

**Who or what inspired you to become a scientist?**

You know how some people know they want to become scientists when they're kids? I was about as far away as you could be. I thought science was pretty dull for most of my childhood, I was much more into creative disciplines – music, theatre, writing, etc. The first time I remember thinking about becoming a scientist was in high school when we learned about Ernest Rutherford and colleagues’ gold foil experiment aimed at deciphering the structure of the atom, and I remember thinking “wow that was such a creative way to figure that out!”. That changed the way I thought about science, but I didn't know any scientists growing up, and I didn't really understand what that career trajectory even looks like. As an undergraduate at Texas Christian University, I was fortunate to have three professors – Mike Chumley, Phil Hartman, and David Minter – who each encouraged and guided me into a career in research.

**What is the main question or challenge in disease biology you are addressing in this paper? How did you go about investigating your question or challenge?**

There's a veterinary sedative called xylazine that, over the past decade or so, has been increasingly found mixed into the illicit opioid supply. When people ingest this xylazine-opioid combination they often develop severe and necrotic cutaneous wounds. Here, we wanted to address the mechanism by which xylazine was causing these wounds. The Huttenlocher lab uses zebrafish models to study wound healing, so I started by seeing what happens to the skin when larvae are exposed to xylazine – we found it disrupted the skin in ways that seemed very similar to in humans. We really had two questions. First, which receptor is xylazine acting through? Xylazine is a well-established alpha-2 adrenergic receptor agonist, but Zoe McElligott's group at UNC-Chapel Hill recently found it also acts on the kappa opioid receptor – and we found that we could replicate the skin effects with kappa opioid receptor agonists but not alpha-2 adrenergic agonists. Second, which cell type is it acting on? The prevailing hypothesis is that xylazine disrupts skin perfusion, causing secondary wounds, but we unexpectedly found that xylazine appeared to act directly on keratinocytes. This was surprising given that single-cell RNA-sequencing databases show near-absent kappa opioid receptor expression in keratinocytes; however, the Human Protein Atlas reveals that keratinocytes are actually among the highest expressors of this receptor at the protein level, suggesting that mRNA is a poor proxy here. Our working model, which needs to be verified in mammalian systems, is that xylazine acts directly on keratinocytes through the kappa opioid receptor to disrupt the skin!

We found that xylazine appears to act directly on skin cells (keratinocytes) to disrupt the skin and, ultimately, cause wounds

**How would you explain the main findings of your paper to non-scientific family and friends?**

The veterinary sedative xylazine has, over the past decade, been increasingly found mixed into the illicit fentanyl and heroin samples across the United States. Xylazine is often referred to as ‘the zombie drug’ because it causes both a pronounced trance-like state as well as severe cutaneous wounds in people that ingest it. How xylazine causes these skin wounds is unknown, which makes them difficult to treat in the clinic. To figure out how xylazine is disrupting the skin in humans, we wanted a way to model these wounds in a laboratory setting. We found that exposing zebrafish larvae to xylazine resulted in a similar kind of skin damage as seen in humans, giving us a way to study how this drug causes wounds in a controlled setting. One of the major hypotheses in the medical literature is that xylazine causes blood vessels in the skin to constrict, resulting in poor oxygenation of the skin, which ultimately results in these wounds. However, we found that xylazine appears to act directly on skin cells (keratinocytes) to disrupt the skin and, ultimately, cause wounds. We hope that these findings will eventually lead to more effective treatments for these skin wounds in the clinic.

**What are the potential implications of these results for disease biology and the possible impact on patients?**

It's my hope that, by figuring out the mechanism by which xylazine causes these wounds, we might be able to design better treatments for these wounds down the road. This paper, building off work from Zoe McElligott's group at UNC-Chapel Hill, raises that possibility that targeting the kappa opioid receptor might have therapeutic value in treating these wounds. Additionally, when you find a drug that causes an unexpected disruption to skin homeostasis, it might be hinting at a role for the kappa opioid receptor and its downstream signalling pathways in normal skin biology. This could open up new lines of inquiry into wound healing more broadly, independent of the xylazine story.

**Figure:**
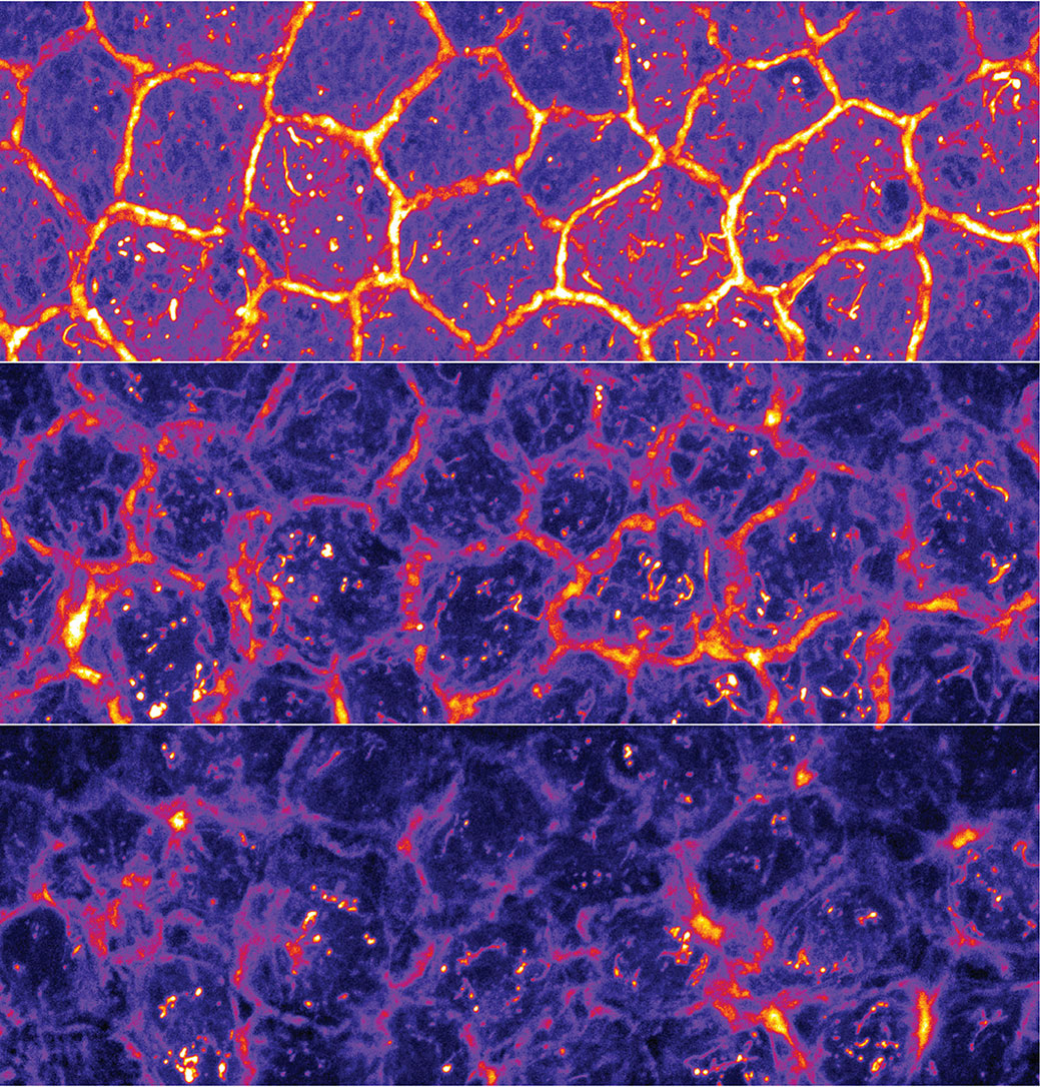
**Timelapse confocal imaging of basal keratinocyte F-actin dynamics in Tg(*krtt1c19e*:LifeAct-mRuby) zebrafish larvae following xylazine exposure, showing progressive disruption of intercellular adhesions and de-adherence of neighbouring cells.** Image acquired by Adam Horn and Tanner Robertson.

**Why did you choose DMM for your paper?**

We're trying to develop a model for a human pathology so we can figure out the mechanism – a journal called Disease Models & Mechanisms seems apt! DMM also has a great reputation for scientific rigour, and a lot of great zebrafish papers get published here.

… one of the most valuable things mentors can do is protect some fraction of their trainees’ time for genuinely risky, exploratory work …

**Given your current role, what challenges do you face and what changes could improve the professional lives of other scientists in this role?**

When you read historical accounts or biographies of science and scientists, it's always striking how so many major discoveries have been either flukes, dumb luck or somebody trying something a little off the beaten path. And yet, I think we often discourage this type of experimentation for our trainees. The combination of funding pressures and the publish-or-perish culture pushes early-career scientists toward safe, incremental projects where the outcome is more predictable – and I think this is a real loss for science. Mentorship culture can compound this problem when trainees are steered away from deviating from the lab's established lines of inquiry. I think one of the most valuable things mentors can do is protect some fraction of their trainees’ time for genuinely risky, exploratory work – the kind of project where you're not sure it'll work, but if it does, it could be really exciting. I've been fortunate to have mentors that have helped me learn how to strike an appropriate balance. This paper was actually a good example of that for me: it was a complete departure from my main research program, but my PI gave me the latitude to pursue it, and it led somewhere interesting.

**What's next for you?**

I am starting my own lab in the Microbiology and Immunology Department at the Cornell University College of Veterinary Medicine this summer. Be on the lookout for more exciting zebrafish research!

**Tell us something interesting about yourself that wouldn't be on your CV**

In college, I played in a few indie rock bands that had a couple songs played on TV shows, including on MTV. If you would like to pay me a fraction of a penny, you can listen to the album Milk & Cereal by Malandros on Spotify.
